# Structured Medication Review and Shared Decision-Making in Patients with Mild Intellectual Disabilities Who Use Psychotropic Medication

**DOI:** 10.3390/pharmacy14010005

**Published:** 2026-01-06

**Authors:** Gerda de Kuijper, Josien Jonker, Rien Hoge

**Affiliations:** 1 Mental Healthcare Drenthe, Department Centre for Intellectual Disability and Mental Health, Middenweg 19, 9404 LL Assen, The Netherlands; josien.jonker@ggzdrenthe.nl; 2 Wilhelmina Hospital Assen, Department Pharmacy, 9401 RK Assen, The Netherlands; rien.hoge@wza.nl

**Keywords:** medication review, shared decision-making, mental health, intellectual disabilities, accessible medication leaflets, psychotropic medication

## Abstract

People with intellectual disabilities frequently use psychotropic and other medications, sometimes inappropriately. To promote shared decision-making, they require accessible information about their medication. This study combined data from two similar intervention studies, conducted in two different settings, to assess the appropriateness of medication use and the shared decision-making process among adults with mild intellectual disabilities who used psychotropic medication. The intervention consisted of a structured, multidisciplinary medication review, including the provision of accessible psychotropic medication leaflets, and a discussion of the pharmacotherapeutic treatment plan with the patient by either a pharmacist or physician, depending on the setting. Outcomes included medication use, pharmacotherapeutic problems, implementation of recommendations, and perceived shared decision-making, measured with the Shared Decision-Making Questionnaire Q9. The 15 included participants used an average of nearly seven medications, which were mainly neurotropic, gastrointestinal, cardiovascular, and respiratory agents. On average, two pharmacotherapeutic problems were identified; the most common were overtreatment, side effects, and administration difficulties. Recommendations often involved dose reduction or tapering, and about 75% were fully or partially implemented. Both participants and clinicians reported high satisfaction with shared decision-making. Multidisciplinary, structured medication reviews, incorporating accessible medication leaflets, may enhance appropriate medication use and shared decision-making, but more research is needed.

## 1. Introduction

Among people with intellectual disabilities, the prevalence of chronic mental and physical health conditions is high [1]. As a consequence, they often have ongoing medication use and are at risk of polypharmacy [2,3]. In particular, psychotropic drugs are frequently prescribed, often long-term, even in children and youth [4,5,6,7,8]. Yet studies among adults and youth in various settings clearly indicate that the prescription of psychotropic medication in particular may be inappropriate [5,7,9,10]. For example, although the National Institute for Health and Care Excellence (NICE) guidelines [11] recommend avoiding the off-label prescription of antipsychotics for challenging behavior, in clinical practice, there may be barriers to discontinuing the long-term use of these agents [12]. This likely explains why, in intellectual disability healthcare, the off-label prescription of antipsychotics for challenging behavior is still common practice. Furthermore, besides the risk of inappropriate prescription, psychotropic medication may cause drug-induced adverse events. Studies in the elderly, adults, and children with intellectual disabilities showed that these populations are vulnerable to side effects of psychotropic medication, which may also have negative consequences for their health and quality of life [2,13,14,15,16,17,18]. Therefore, it is important to carefully weigh the advantages and disadvantages in decisions about new or ongoing treatments with psychotropic medication for mental health conditions in people with intellectual disabilities. This may be even more important in the case of treatments for challenging behavior, since the effectiveness of psychotropic medication in this indication has not been proven and non-pharmacological treatments are recommended [11].

The monitoring and evaluation of the effects of psychotropic and other medications in people with intellectual disabilities varies across and within countries, due to differences in healthcare [19,20,21,22,23]. In the Netherlands, care depends on living arrangements: those in residential facilities generally receive healthcare according to the Long-Term Care Act, while those in community settings rely on primary care. Monitoring of the use of medication in residential settings is often performed by nurses, with yearly medication reviews by affiliated pharmacists and intellectual disability physicians, whereas, in community settings, it is the responsibility of GPs or specialized healthcare. However, in the case of psychotropic medication, GPs may feel not fully equipped to do so, and there is a risk that monitoring will not take place at all [24,25]. In this patient group, regular medication reviews are often lacking in both primary care and specialized mental healthcare.

**Figure 1:**
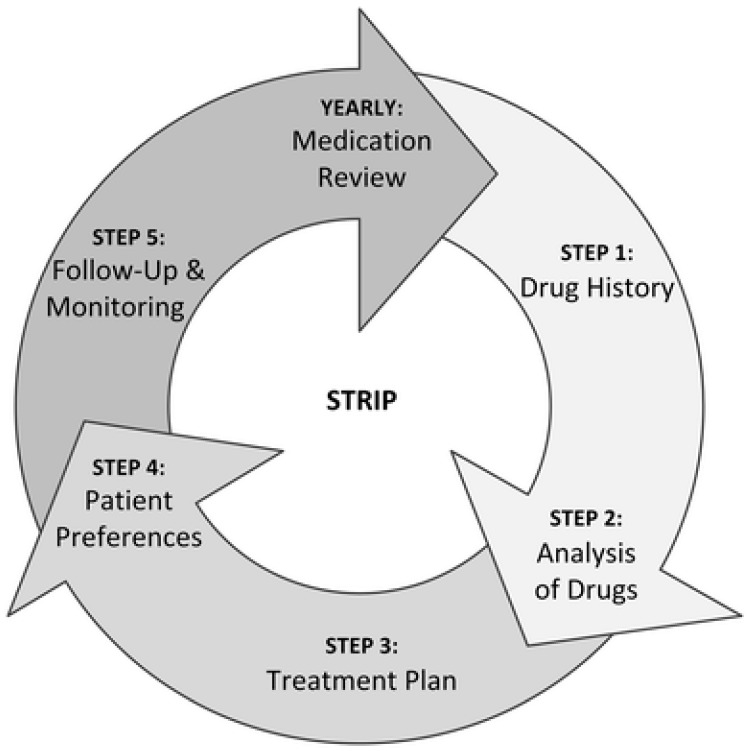
Systematic Tool to Reduce Inappropriate Prescribing (STRIP) method [33].

Medication reviews can help to identify inappropriate prescriptions, to improve prescription practice, and to optimize pharmacotherapy. Especially in vulnerable patient groups, like the elderly and people with intellectual disabilities who use psychotropic medication, medication reviews may be needed because of increased risks for adverse events, such as the occurrence of harmful side effects and interactions with other medications used in this often overmedicated population. Indeed, a focused medication review study on psychotropic drug prescribing yielded 26 studies conducted in the elderly, dementia, and intellectual disability populations, which all reported changes in psychotropic drug prescribing [26]. The results of an additional study indicated the feasibility of delivering structured psychotropic medication reviews in community services for people with intellectual disabilities in the United Kingdom [27]. In a systematic review, including studies in acute elderly care and long-term elderly care group homes, it was found that structured medication reviews with the help of a screening tool for inappropriate prescribing could reduce in inappropriate prescriptions [28]. Furthermore, two other systematic reviews on pharmacist-led interventions such as medication reviews—one in primary care among older adults and one in community care among patients with a range of clinical conditions and ages—showed an improvement in medication appropriateness [29,30]. Moreover, in intellectual disability healthcare, studies on medication reviews have been conducted. The quality of pharmacotherapy in patients with intellectual disabilities who received home care improved after interdisciplinary medication reviews [10]. In a systematic review and meta-analysis of sixteen pre–post studies on interventions to reduce inappropriate psychotropic drug prescription in people with neurodevelopmental disabilities, it was found that medication reviews, multicomponent interventions, workplace training, and guidelines were all effective [31]. However, in a recent Cochrane systematic review regarding studies on appropriate medication use in the elderly, it was concluded that it was unclear whether interventions resulted in significant improvements in appropriate polypharmacy [32]. Nonetheless, it appears that, since 2018, the number of studies on interventions to address potential prescribing omissions has been increasing and that more interventions are being delivered by multidisciplinary teams [32]. The Systematic Tool to Reduce Inappropriate Prescribing (STRIP) method is one of these multidisciplinary interventions. It consists of five steps: a medication assessment, a pharmacotherapy review, a pharmacotherapeutic treatment plan, shared decision-making, and follow-up and monitoring (see Figure 1) [33]. Studies show that this method is feasible in primary care [34]. A pilot study, using the Systematic Tool to Reduce Inappropriate Prescribing (STRIP) method in 27 adults with intellectual disabilities in three residential settings, revealed 127 drug-related problems, mostly consisting of the use of unnecessary medications [35]. However, in these settings, after six months, just 15.7% of the recommended interventions were implemented [35].

In shared decision-making on medical treatments, information exchange should be bidirectional and include medical information as well as patient preferences. In treatment decisions, the perspectives of the treating clinician and the patient should be equally important. These two parties work together in a process of shared decision-making by weighing the advantages and disadvantages of treatment options suited to the patient’s preferences and values. Hence, clinicians should ensure that information about the advantages and disadvantages of treatment options is understood by their patients. This is especially important with regard to medication treatments, since treatment adherence is a prerequisite for treatment effectiveness and knowledge about the prescribed medication may decrease the risk of drug-induced adverse events. Indeed, in a systematic review on treatment adherence among individuals with mental disorders, it was found that shared decision-making could help to increase treatment adherence [36]. However, the provision of understandable information about medication use may be insufficient for people with intellectual disabilities and/or low levels of literacy [37,38,39]. Furthermore, people with intellectual disabilities often need support from paid caregivers and/or relatives in treatment decisions. Thus, there is a need to involve these stakeholders in the provision of information about treatments with psychotropic medication, shared decision-making, and treatment evaluations [39]. Here, it is also important to ensure that understandable information is provided, since a number of studies have shown that patients as well as their caregivers are insufficiently informed about medication use by their doctor or pharmacist [40,41,42,43,44]. Accessible medication information is also a prerequisite to ensure that the information exchange between the patient with an intellectual disability and the clinician is effective, and, in this way, it is possible to increase patients’ autonomy in decision-making and self-determination regarding medication use.

People with intellectual disabilities wish to be involved in monitoring, evaluation, and shared decision-making regarding their psychotropic drug use [39,40,42]. Since the STRIP method includes an evaluation of treatment effects and a shared decision-making step, this type of medication review may increase their satisfaction with their involvement in this regard. To our knowledge, there are no studies combining these two issues. The aim of the present study is to explore the results of structured medication reviews and shared decision-making, with the help of accessible medication information, in individuals with intellectual disabilities in community settings who use psychotropic medication. We aimed to investigate the number and types of pharmacotherapeutic problems, the content and implementation of the pharmacotherapeutic plan, and satisfaction with shared decision-making among prescribers and patients regarding medication use.

## 2. Materials and Methods

### 2.1. Study Design and Setting

For this study, we aggregated data from two similar pilot studies on shared decision-making in psychotropic drug treatment in patients with intellectual disabilities. The studies were conducted in the northeast of the Netherlands in an intellectual disability specialized mental healthcare center (setting 1) and in primary healthcare settings (setting 2) from 1 September 2022 to 1 May 2024 and 1 September 2023 to 1 May 2025, respectively.

The two pilot studies included an intervention, i.e., a structured medication review according to the STRIP method, which we slightly adapted to fit within the working processes of each setting. The medication review consisted of

(1) A medication consultation by a nurse (setting 1) or pharmacist (setting 2), including the pharmacotherapeutic anamnesis, the assessment of side effects, and the provision and discussion of accessible medication leaflets on the type of psychotropic medication that the participant was using;
(2) A medication review, including a pharmacotherapeutic analysis using an assessment form (see Section 2.4 for explanation) and the drawing up of a pharmacotherapeutic treatment plan by the pharmacist and doctor (setting 1) or the pharmacist alone (setting 2), which was subsequently discussed with the participant and/or participants’ relatives by the doctor (setting 1) or pharmacist (setting 2);
(3) A follow-up after six months.

In Table 1, the operationalization of the structured medication review performed in this study is outlined.

### 2.2. Participants

Participants could be included in the pilot intervention studies when they used one or more psychotropic drugs and received care from the specialized mental healthcare outpatient clinic (setting 1) or from a regional home care organization in addition to care from their general practitioner (GP) and community pharmacist (setting 2). All patients in the mental healthcare setting were known to have an intellectual disability and all patients in the home care organization were known to have an intellectual disability, autism, and/or psychiatric and social vulnerabilities.

Recruitment in the mental healthcare setting took place among respondents to a previous survey, which was conducted from 1 January to 31 December 2022 in the same setting [39]. Additionally, clinicians from this setting were asked to recruit participants among their new patients who met the eligibility criteria of the study.

Recruitment in the primary care setting took place by sending a letter with accessible information about the study and an invitation to participate to all patients of the home care organization in the province of Drenthe and adjacent areas.

In both settings, patients who had indicated that they were interested were contacted by a research assistant, by phone and/or mail, to determine whether they met the inclusion criteria. Those who appeared eligible were invited for a personal consultation, during which the study was explained in more detail with the help of accessible leaflets about the study and an accessible informed consent form. From those who agreed to participate and gave their informed consent, either directly or via their legal representatives, inclusion could start. In the primary care setting, following consent, participants’ GPs and pharmacists were also contacted to ask for their collaboration in their patients’ intervention (i.e., the medication consultation and review) and their own commitment and consent to participate in the study for data collection. We also asked GPs to confirm whether participants had an intellectual disability—or, if this was not registered, whether their patients had adaptive and cognitive disabilities, similar to the level of a mild intellectual disability.

One GP did not consent to participate, so, finally, 10 participants started the study in primary care; all had a known mild intellectual disability or known similar disabilities in cognitive and adaptive functioning. However, for three of these ten participants, data on medication use at the time point of the medication review were missing.

**Figure 2:**
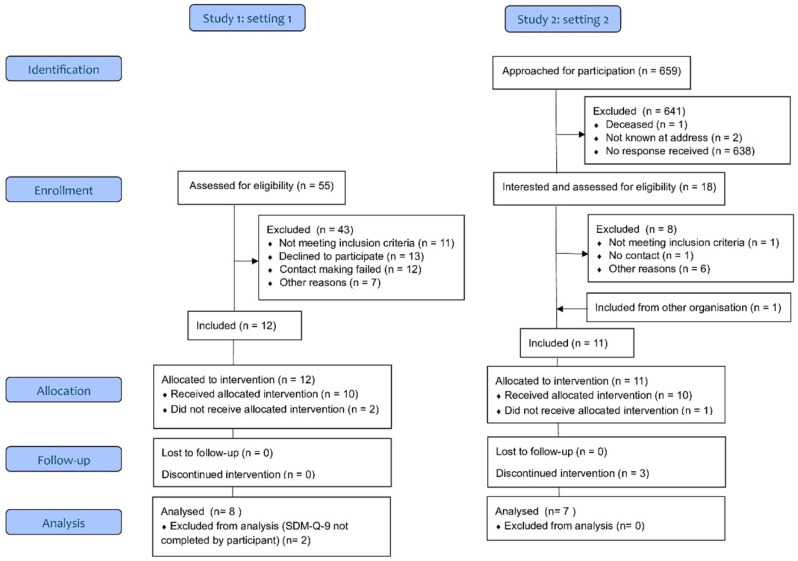
Flowchart of participants in the convenience sample.

In the present study, we included only participants who had completed the SDM-Q-9 (see Section 2.4) by themselves and for whom medication use at the time point of the medication review was known. This was the case in 8 of the 12 included participants in the specialized mental healthcare setting and in 7 of the 10 in the primary care setting. Thus, for the present study, we obtained a convenience sample of 15 participants in total from the two pilot studies (see Figure 2).

### 2.3. Ethical Issues

The study protocols of both studies were submitted to the Medical Ethics Review Board of the University Medical Centre Groningen, The Netherlands (METc2021/591 and METc2022/391). The board concluded that the research did not fall within the scope of the Dutch Medical Research Involving Human Subjects Act. Both studies were conducted according the European General Data Protection Regulation and Dutch professional guidelines regarding healthcare research. Data were pseudonymized during data collection by using a code list, which was only accessible by the principal investigator and authorized study delegates. Data were stored at the mental healthcare setting according to the strict security and back-up policy of this setting.

All subjects and/or their legal representatives received verbal and written accessible explanations and information. All signed an informed consent form confirming that they agreed to participate voluntarily in the study and that their data could be used anonymously. Subjects could stop their participation in the study at any moment and without mentioning a reason.

### 2.4. Materials

In both pilot studies, we used the Shared Decision-Making Questionnaire (SDM-Q-9) to assess satisfaction with shared decision-making regarding medication use. This questionnaire consists of two versions, each with nine questions regarding the various aspects that determine satisfaction with shared decision-making in treatment decisions (see Table 2). The patient version should be completed by the patient and the practitioner version by the clinician who had discussed the advantages and disadvantages of the specific treatment options with the patient.

The SDM-Q-9 is a validated instrument, widely used in healthcare and research [45,46]. It should be rated on a six-point Likert scale from completely disagree (score 1) to completely agree (score 6). Higher scores represent greater satisfaction with the shared decision-making process. In the pilot studies, the SDM-Q-9 was completed by the two parties involved in shared decision-making, i.e., the participant and/or participant’s relative and the doctor (setting 1) or pharmacist (setting 2). The SDM-Q-9 patient version was made accessible by adding an introduction in easy-to-read language, a link to a video with a spoken version of the questionnaire, and smileys for the rating scale.

Medication side effects were assessed with the Matson Evaluation of Side Effects Scale (MEDS) [47]. This questionnaire consists of 90 items in total, which should be rated by a competent healthcare professional for presence and severity (0 = not present/no problem, 1 = mild or moderate problem, 2 = serious problem) and, in the case of presence, for their duration (1 ≤ 1 month, 2 = 1–12 months, 3 ≥ 12 months, or don’t know). In this study, the MEDS was completed by the nurse (setting 1) or pharmacist (setting 2). There are nine subscales: cardiovascular and hematologic effects; gastrointestinal effects; endocrine and genitourinary effects; eye, ear, and throat effects; skin, allergies, and temperature; and four subscales regarding the neurological effects of psychotropic medication on the central nervous system (CNS)—CNS general, CNS dystonia, CNS parkinsonism/dyskinesia, and CNS behavior/akathisia.

To explain and discuss the advantages and disadvantages of psychotropic medication, we developed 64 accessible medication leaflets on a wide range of psychotropic drugs [48].

Finally, we created an assessment form to categorize the pharmacotherapeutic problems. The categories included undertreatment, not effective, overtreatment, (potential) side effects, contraindication, inappropriate dosage, and administration problems. In both settings, this form was completed by the pharmacist.

### 2.5. Data Collection

Data included participants’ demographic characteristics, medication use at the time of the medication review, the pharmacotherapeutic analysis and treatment plan, outcomes of the SDM-Q-9 completed by both participants and clinicians immediately after the medication review, and data on the completion of the pharmacotherapeutic interventions as advised in the pharmacotherapeutic treatment plan six months after the medication review. Medication use was categorized according to the Anatomic Therapeutic Chemical (ATC) class. Outcomes were the number and types of medications, side effects, the number and types of pharmacotherapeutic problems, the content and implementation of the pharmacotherapeutic plan, and satisfaction with shared decision-making among prescribers and patients regarding medication use. With regard to side effects, we used only the data/ratings from the four CNS MEDS subscales and only the severity ratings (40 items in total), since the focus of the present study was on psychotropic medication.

### 2.6. Statistical Analyses

We used IBM SPSS Statistics Version 30 for the descriptive analyses (frequencies and means) of the quantitative data. We used the nonparametric Wilcoxon rank sum test (also known as the Mann–Whitney U test for independent samples) to investigate potential differences between settings and groups. Spearman’s correlation was used to investigate the potential correlations between the number of medications used and the number of pharmacotherapeutic problems.

## 3. Results

### 3.1. Participant Characteristics

In Table 3, the demographic characteristics, the total number and types of medication used, and the severity of neurological side effects among participants are shown.

The mean number of medications used by the 15 participants was 6.7 (range 2–14). Polypharmacy (use of >5 agents simultaneously) was present in nine participants (60%). The most frequently used class of medication was ATC.N (neurological system medication, including psychotropic medication). At the time point of the medication review, 93% of participants were using this type of medication. One participant (7%) had stopped the use of methylphenidate after study inclusion but before the medication review. The mean number of psychotropic agents in these 93% of patients was 2.9. Other frequently used classes of medication were ATC.A (gastrointestinal/metabolism medication, 66% of participants, mean number 1.7), ATC.C (cardiovascular system, 47% of participants, mean number 1.9), and ATC.R (respiratory system, 47% of participants, mean number 1.4).

We found no correlation between the number of medications used and the number of identified pharmacotherapeutic problems, and there were no differences regarding these variables between the participants in the mental healthcare setting and those in the primary care setting.

### 3.2. Psychotropic Medication Use and Review

Table 4 shows the types of psychotropic medication used by the participants, the total number and types of pharmacotherapeutic problems, the pharmacotherapeutic plan, and the degree and type of implementation of the plan six months after the medication review.

Two thirds of participants used antipsychotics, more than half used antidepressants, almost half used hypnotics/sedatives, and more than a quarter used mood stabilizers. Regarding the severity of neurological side effects, the data of 10 participants were available (Table 2). Of these, nine experienced one or more side effects (mean score 9.9, range 2–19).

A medication review could be conducted in 12 of the 15 participants. Among these 12, the mean number of pharmacotherapeutic problems identified was 1.8 (range 0–4). In 10 of these 12 cases, at least one pharmacotherapeutic problem was present. The most frequently observed issue was overtreatment, occurring in 90% of these cases. Other common problems included (potential) side effects (40%) and administration issues (30%).

Data on the completion of the proposed interventions were missing for 3 of the 15 participants. Among the remaining 12, interventions were (partially) completed in nine participants.

### 3.3. Shared Decision-Making Comparisons Between Participants’ Ratings and Clinicians’ Ratings by Setting

**Figure 3:**
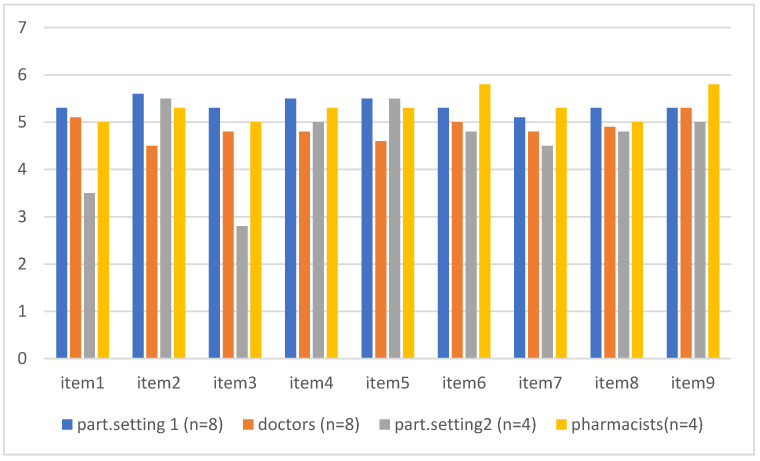
Mean scores for ratings on a Likert scale ranging from 1 (completely disagree) to 6 (completely agree) by item of the SDM-Q-9 questionnaire. This measure was completed by the two parties in the shared decision-making process regarding pharmaceutical treatments, i.e., patient and doctor in setting 1 (specialized mental healthcare) and patient and pharmacist in setting 2 (primary care).

In Figure 3, the mean scores by item of the SDM-Q-9 are shown for the two parties involved in SDM, i.e., patients and clinicians. In this study, these parties were the participants and their prescribing doctors in the mental healthcare setting (dark blue and orange bars) and the participants and pharmacists in the primary care setting (grey and yellow bars).

With regard to the total score on the SDM-Q-9, participants in the mental healthcare setting had higher mean ratings than their doctors (mean = 48 and mean = 44, respectively; *p* = 0.05, Wilcoxon W = 49). At the item level, participants in this setting rated higher than their doctors for item 2 (My doctor wanted to know exactly how I wanted to be involved in SDM; mean rating = 5.5 versus 4.5, *p* = 0.05, Wilcoxon W = 49) and item 4 (My doctor precisely explained the advantages and disadvantages of the treatment options; mean rating = 5.6 versus 4.6, *p* = 0.02; Wilcoxon W = 45.5). In the primary care setting, there were no differences between the participants and their pharmacists with regard to the SDM-Q-9 total score, including at the item level.

### 3.4. Comparisons Between Settings

When comparing the clinicians between the settings, the mean SDM-Q-9 ratings of the pharmacists (primary care) were higher than the ratings of the doctors (specialized mental healthcare) (mean = 47.5 and 43.6, respectively; *p* = 0.001, Wilcoxon W = 40.5). When comparing the participants between the settings, there were no differences in ratings for the SDM-Q-9 total or at the item level.

## 4. Discussion

In this study, we explored medication use, the outcomes of medication reviews, and satisfaction with shared decision-making regarding medication use in patients with a mild intellectual disability or similar levels of functioning in community healthcare settings. We included fifteen participants who used at least one psychotropic agent. In these fifteen, the number of prescribed agents varied from 2 to 14 and, in nine, polypharmacy (use of >5 agents) was present. Following psychotropics, gastrointestinal/metabolism, cardiovascular, and respiratory system medications were frequently prescribed (in 66%, 47%, and 47% of participants, respectively).

Our findings are in line with those of O’Dwyer et al. (2016), who investigated the medication patterns of a representative sample of 753 adults and elderly individuals with intellectual disabilities [49]. Here, also, after neurological system medication, gastrointestinal and cardiovascular medications were the most frequently prescribed drug classes, and polypharmacy was also present in more than half of the participants. The medication patterns that were found in the large-scale study of O’Dwyer et al. (2016) and in our small-scale study may highlight the increased risk of co-occurring physical disorders alongside mental health issues in the intellectual disability population [1], as well as in the general population [50,51]. Given the heightened health risks associated with psychotropic medications, including vulnerability to adverse drug reactions and interactions, the importance of medication reviews in individuals with mental disorders cannot be overstated [49].

A medication review was performed for twelve of the fifteen participants. Among these twelve, overtreatment was identified in nine, while no pharmacotherapeutic problems were found in two. The corresponding pharmacotherapeutic plans often included dose lowering or tapering of proton pump inhibitors and/or sedatives and antipsychotics. Administration problems were present in three and (a risk of) side effects in four participants. In these cases, the pharmacotherapeutic plans mostly included changes in the intake schedule and referral to GPs or medical specialists for changes in the type of medication and/or the monitoring and handling of side effects. Patients were also referred to their GPs or specialists to handle undertreatment and sometimes overtreatment with asthma or cardiovascular medication.

The mean number of pharmacotherapeutic problems that was found in our study in the community setting is lower than that in the study of Zaal et al. (2016) in a residential setting [35] (1.8 versus 4.7). However, their finding of potentially inappropriate or unnecessary drug use (i.e., overtreatment) as the most frequent problem is similar to the findings of our study. In nine of the twelve participants in our study, the pharmacotherapeutic plans were (partially) implemented, which was much higher than in the study of Zaal et al. (2016), including a sample of 27 participants (75% versus 16%) [35]. The higher rate of successful implementation in our study could be related to the involvement of the patients themselves in the discussion with the pharmacist or doctor about the pharmacotherapeutic treatment plan. Setting characteristics may also have played a role (institutional setting versus community setting).

The findings of dose reductions for psychotropic medication after medication review are in line with the focused psychotropic medication review study of Sheehan et al. (2018) [26] and may confirm the hypothesis of the risk of suboptimal drug therapy in the case of patients with psychotropic drug use. Conditions that may enable successful psychotropic deprescribing were mentioned by pharmacists in a Delphi study on this topic [52] and included the involvement of patients and/or their caregivers, a multidisciplinary approach, education and knowledge regarding medication use, good communication, and clear documentation. These conditions were largely present in our study, e.g., education and knowledge about medication use by the provision of accessible leaflets. Furthermore, in our study, dose lowering or cessation of proton pump inhibitors was often advised and successfully implemented, which is in line with other studies that revealed potential inappropriate prescribing of these agents and successful deprescribing after medication review [53,54].

We also assessed the satisfaction of the patient and the clinician with the shared decision-making process in the discussion about the pharmacotherapeutic treatment plan. As measured with the SDM-Q-9, participants and their clinicians were satisfied with the process. It is likely that the provision of accessible medication leaflets and the discussion of participants’ own experiences and beliefs regarding medication effects during the medication consultation and subsequent discussions of treatment plans contributed to the great satisfaction with the shared decision-making process in both parties. Remarkably, in the specialized mental healthcare setting, doctors rated somewhat lower than their patients, while this was not found in the community care setting. This may have been caused by differences between the specialized doctors and community pharmacists regarding communication skills and the understanding of people with intellectual disabilities. Due to the greater expertise of the former group, they likely had a better understanding of the pitfalls of a shared decision-making process in this patient group, e.g., the tendency for socially desirable responding. Our findings may confirm other findings from the literature that multidisciplinary pharmacy-led interventions are effective in reducing inappropriate prescribing, especially when patients themselves are involved in decision-making [10,36,37,43]. The STRIP method used in our study is multidisciplinary; patients were involved in decision-making and treatment plans were, to a large extent, implemented.

A limitation in this study is that we relied solely on clinicians’ information regarding whether participants had intellectual disabilities or functioned at similar levels. Furthermore, selection bias possibly played a role. Our sample included 15 participants derived from the two pilot studies. Recruitment for the two pilot studies took place largely among patients who were interested in the subject. Thus, the present sample might not be representative of people with intellectual disabilities who use psychotropic medication and receive community healthcare. Therefore, we cannot conclude that the medication patterns that we found are similar to those that were found in the representative study of O’Dwyer et al. (2016) [49]. Furthermore, given the significantly higher ratings on the SDM-Q-9 of the patients in the mental healthcare setting compared to their doctors, participants in this setting could have tended to provide socially desirable answers. Relationships between patients and doctors are characterized by a greater dependency than those between patients and pharmacists in the primary care setting. However, these findings should be interpreted in light of the small sample size. Further research, especially qualitative research, is needed to investigate how satisfaction with shared decision-making in structured medication reviews could best be measured in different settings.

Despite these limitations, this study provides a picture of the medication patterns, medication problems, the implementation of advised medication changes, and satisfaction with shared decision-making among people with intellectual disabilities who receive community healthcare.

Although we did not assess the effects of the provision of accessible medication leaflets, we presume that the use of these leaflets during the review process contributed to the great satisfaction with the shared decision-making process. Furthermore, we did not investigate the effects of this type of medication review on the prescription practice in people with intellectual disabilities in community healthcare. Studies in this population are needed to investigate the effects on appropriate prescribing, shared decision-making, adverse events, and treatment adherence and the cost-effectiveness of STRIP-wise medication reviews with the use of accessible medication leaflets.

## 5. Conclusions

A multidisciplinary systematic medication review with the use of accessible psychotropic medication leaflets in a small sample of patients with intellectual disabilities in community healthcare led to a high level of implementation of treatment advice to increase appropriate medication use. Both patients and clinicians were highly satisfied with the process of shared decision-making. Further research is needed regarding the effectiveness of this intervention for appropriate medication use and on satisfaction with shared decision-making in medication use among this population.

## Figures and Tables

**Table 1:** Operationalization of the structured medication review.

Step 1 ^#^ Medication consultation	Medication history (medical record) Current medication regimen Assessment of medication management Arranging laboratory tests (Arranging) physical examinations Assessment of side effects Providing and discussing accessible information on the type of psychotropic medication(s) the participant uses
Step 2 ^#^ Medication review	(a) Pharmacotherapeutic analysis: evaluation of the current medication regimen - Undertreatment: missing necessary medication - Ineffective treatment: whether treatment goals were being met - Overtreatment - Presence of adverse effects or symptoms that could potentially be explained by the current medication use - Assessing laboratory results and physical examination findings: abnormalities that may be related to medication use - Other medication-related issues identified at step 1: correct use, appropriateness of formulation or dosage form, medication taken at appropriate time of day - Examining possible contraindications, conflicting therapies, and potential drug interactions (b) Formulating a draft treatment plan - Addressing potential pharmacotherapeutic problems - Determining overall treatment goals in relation to the identified issues and proposed interventions, e.g., tapering or discontinuing medication or adjusting dosages (c) Discussing the draft treatment plan with the participant and/or participants’ relatives in a shared decision-making process, leading to a treatment plan endorsed by all parties
Step 3 ^#^ Follow-up	Evaluating the outcomes of the interventions outlined in the treatment plan

**^#^** In specialized mental healthcare (setting 1), step 1 is conducted by a nurse, step 2a by the pharmacist, step 2b by the pharmacist and the prescribing physician, step 2c by the physician, and step 3 by the nurse.

**Table 2:** Content of the Shared Decision-Making Questionnaire (SDM-Q-9) on medical treatments. Each question should be answered by the two parties in the shared decision-making process, i.e., the clinician (in this study, the doctor or pharmacist) and the patient.

Item	Statement
1	My doctor/pharmacist made clear/I made clear that a decision needs to be made.
2	My doctor/pharmacist wanted to know exactly/I wanted to know exactly from my patient how I/he/she want(s) to be involved in making the decision.
3	My doctor/pharmacist told me/I told my patient that there are different options for treating my/his/her medical condition.
4	My doctor/pharmacist/I precisely explained the advantages and disadvantages of the treatment options (to my patient).
5	My doctor/pharmacist/I helped me to understand/my patient understood all the information.
6	My doctor/pharmacist/I asked me/my patient which treatment option I/he/she prefer(s).
7	My doctor/pharmacist/my patient and I thoroughly weighed the different treatment options.
8	My doctor/pharmacist/my patient and I selected a treatment option together.
9	My doctor/pharmacist/my patient and I reached an agreement on how to proceed.

**Table 3:** Participant characteristics at the time point of medication review.

Participant	Setting ^1^	Gender	Age (Years)	Medication Total Number	Number by ATC Class ^2^ of Prescribed Medication										N.S. ^3^
					A	B	C	D	G	L	M	N	R	S	
1	2	female	30	7	1				1		1	4			10
2	2	female	50	7	3	1						2	1		m ^5^
3	2	male	67	10	3	1	1					4	1		m
4	2	female	50	6	1		1		1			3			m
5	2	female	61	10	3	1	3			1		2			5
6 ^4^	2	female	47	9	1	2	2		1				1	2	m
7	2	male	36	2								1	1		m
8	1	male	56	9	1		4					3	1		3
9	1	female	33	14	2	1	1		1			5	4		15
10	1	female	36	3	1							2			19
11	1	female	34	5							1	4			0
12	1	female	25	2	1							1			19
13	1	female	35	6								6			8
14	1	male	36	5	2							3			2
15	1	male	38	7	1		1	1				2	1	1	8

^1^ 1 = Specialized mental healthcare, 2 = primary care. ^2^ Anatomic Therapeutic Chemical class. A = alimentary tract and metabolism; B = blood and blood-forming organs; C = cardiovascular system; D = dermatologicals; G = genitourinary system and sex hormones; L = antineoplastic and immunomodulating agents; M = musculoskeletal system; N = neurological system; R = respiratory system; S = sensory organs. ^3^ N.S. = total score of neurological side effects as measured with the Matson Evaluation of Side Effects Scale (MEDS), subscales: central nervous system, dystonia, parkinsonism/dyskinesia and behavior/akathisia; maximum total score = 82. ^4^ Participant 6 used methylphenidate at the time point of inclusion in the study but had stopped use at the time point of systematic medication review. ^5^ m = missing, because the MEDS was not completed.

**Table 4:** Psychotropic medication use, number and types of pharmacotherapeutic problems, content of the pharmacotherapeutic treatment plan, and degree and type of implementation of the pharmacotherapeutic plan and changes in medication six months after the medication review.

Participant	Type of Psychotropic Medication	Number of Pharmacotherapeutic Problems	Pharmacotherapeutic Problems ^1^	Pharmacotherapeutic Plan	Degree ^2^ and Type of Implementation of Pharmacotherapeutic Plan/Change in Medication
1	Antipsychotic Antidepressant Hypnotic/sedative	Missing ^&^	Missing ^&^	No change	Dose increase quetiapine because of psychiatric state
2	Antipsychotic Antidepressant	3	Overtreatment Inappropriate dosage Administration problems	Dose reduction pantoprazole, ferrous fumarate, and quetiapine Folic acid + calcium supplementation	Yes, but attempted dose reduction of quetiapine was not successful Yes
3	Antipsychotic Hypnotic/sedative	3	Not effective	Change in type of sleep medication and asthma medication	Yes
			Overtreatment	Change intake schedule anti-diabetic medication	Yes
			Administration problems	Tapering and stop pantoprazole	Yes
4	Antidepressant Mood stabilizer	Missing ^&^	Missing ^&^	Missing ^&^	Missing ^&^
5	Antidepressant Mood stabilizer	1	Undertreatment	Change intake schedule medication	Yes
				Side effect monitoring (blood pressure)	Yes
6 ^3^	None	0	None	Participant had decided by herself to stop the use of methylphenidate because of no effect No further actions are required	Missing ^&^
7	Antipsychotic	Missing ^&^	Missing ^&^	Missing ^&^	Missing ^&^
8	Antipsychotic Antidepressant	3	Undertreatment Overtreatment (Potential) side effects	No change in psychotropic medication	Yes
				Referral to medical specialist for change in asthma and cardiovascular medication	Yes
9	Antipsychotic Hypnotic/sedative Melatonin (sleep medication)	1	Overtreatment	No change in psychotropic medication. Consider to stop melatonin	Partially
				Referral to general practitioner because of overtreatment asthma medication	Yes, but no change in asthma medication
10	Antidepressant Hypnotic/sedative	1	Overtreatment	Stop melatonin Dose reduction omeprazole	No No
11	Antidepressant Hypnotic/sedative	2	Overtreatment (Potential) side effects	Referral to general practitioner because of potential side effects and interactions	Yes
				Consider dose reduction temazepam/diazepam	Yes; dose reduction temazepam and diazepam
				(Pharmaceutical) support to quit smoking	Partially
12	Antipsychotic	1	Overtreatment	Tapering and stop omeprazole	Partially; dose reduction omeprazole
13	Antipsychotic Antidepressant Hypnotic/sedative Mood stabilizer	4	Not effective Overtreatment (Potential) side effects Administration problems	Tapering pantoprazole	No
				Tapering lorazepam and dose reduction haloperidol	Yes
				Start levothyroxine	Yes
14	Antipsychotic Hypnotic/sedative	0	None	Referral to general practitioner because of side effect monitoring (blood pressure and body weight)	Unknown
				Consider tapering olanzapine	Partially, but dose increase for olanzapine
15	Antipsychotic Mood stabilizer Acramprosate	3	Overtreatment - (Potential) side effects - Contraindication	Referral to general practitioner because of indication for and potential side effects of asthma medication	No, not yet
				Consider changing or stopping pimozide because of potential contraindication Parkinson’s disease and use of L-dopa	Yes, complete discontinuation of pimozide

^1^ Established pharmacotherapeutic problems after the pharmacotherapeutic analysis of medication use. ^2^ Yes = completely implemented, No = not implemented, Partially = not all advised interventions are implemented. ^3^ Participant 6 used methylphenidate at the time point of inclusion in the study but had stopped use at the time point of systematic medication review. ^&^ No information available because the assessment of medication use did not take place at this time point.

## Data Availability

The raw data supporting the conclusions of this article will be made available by the authors on request.
